# Ponatinib: A Review of the History of Medicinal Chemistry behind Its Development

**DOI:** 10.3390/ph17101361

**Published:** 2024-10-11

**Authors:** Mayara Nascimento, Stefany Moura, Lidia Parra, Valeska Vasconcellos, Gabriela Costa, Debora Leite, Maria Dias, Tácio Vinício Amorim Fernandes, Lucas Hoelz, Luiz Pimentel, Monica Bastos, Nubia Boechat

**Affiliations:** 1 Programa de Pós-Graduação em Farmacologia e Química Medicinal do Instituto de Ciências Biomédicas–ICB-UFRJ, Centro de Ciências da Saúde-CCS, Bloco J, Ilha do Fundão, Rio de Janeiro 21941-902, RJ, Brazil; mayarasalles6@gmail.com (M.N.); stefanybazan6@gmail.com (S.M.); 2 Departamento de Síntese de Fármacos, Instituto de Tecnologia em Fármacos, Farmanguinhos–Fiocruz, Manguinhos, Rio de Janeiro 21041-250, RJ, Brazil; valeskaladi@gmail.com (V.V.); gabriela.icfio@gmail.com (G.C.); debora.inacio@live.com (D.L.); mariaconcz@hotmail.com (M.D.); tacio.fernandes@fiocruz.br (T.V.A.F.); luiz.pimentel@fiocruz.br (L.P.); monica.bastos@fiocruz.br (M.B.); 3 Programa de Pós-Graduação Acadêmico em Pesquisa Translacional em Fármacos e Medicamentos–Farmanguinhos, Fundação Oswaldo Cruz, Rio de Janeiro 21041-250, RJ, Brazil; lidiacparra@gmail.com; 4 Laboratório Computacional de Química Medicinal—LCQM, Instituto Federal do Rio de Janeiro—IFRJ, Campus Pinheiral, Pinheiral 27197-000, RJ, Brazil; lucas.hoelz@ifrj.edu.br

**Keywords:** tyrosine kinase inhibitor, dual inhibitors, leukemia, DFG-out, T315I, ponatinib

## Abstract

The primary treatment for chronic myeloid leukemia (CML) involves first- and second-generation tyrosine kinase inhibitors (TKIs), such as imatinib, nilotinib, bosutinib, and dasatinib. However, these medications are ineffective against mutations in the kinase domain of the ABL1 protein, particularly in the protein with the T315I mutation. To address this, ponatinib (PNT), a third-generation inhibitor, was developed. Despite its efficacy in treating the BCR-ABL1^T315I^ mutation, the use of PNT was briefly suspended in 2013 due to serious adverse effects but was subsequently reintroduced to the market. During the drug discovery and development process, it is rare to consolidate all information into a single article, as is the case with ponatinib. This review aims to compile and chronologically organize the research on the discovery of ponatinib using medicinal chemistry tools and computational methods. It includes in silico calculations, such as the octanol/water partition coefficient (cLogP) via SwissAdme, and 2D maps of intermolecular interactions through molecular docking. This approach enhances understanding for both specialists and those interested in medicinal chemistry and pharmacology, while also contextualizing future directions for further optimizations of ponatinib, facilitating the development of new analogs of this crucial inhibitor for the treatment of CML and Philadelphia chromosome-positive acute lymphoblastic leukemia (ALL).

## 1. Introduction

Chronic myeloid leukemia (CML) is characterized by reciprocal translocation between chromosomes 9 and 22, resulting in the formation of a shortened chromosome known as the Philadelphia (Ph) chromosome [1]. This chromosome encodes a constitutively active cytoplasmic tyrosine kinase, BCR-ABL1 (breakpoint cluster region-Abelson 1 leukemia) [2], the main molecular target of CML [3]. Under normal conditions, the ABL1 protein regulates actin remodeling, adhesion, cell motility, and responses to DNA damage and pathogens. Its dysregulation is linked to several cancers, including CML, where *ABL1* fuses with *BCR* due to chromosomal translocation, generating an oncogenic fusion protein [4].

Since the introduction of imatinib (IMT), the first commercial tyrosine kinase inhibitor (TKI) for BCR-ABL1, in 2001, there has been a revolution in the treatment of CML, achieving remarkable results [5]. However, more than half of patients treated with IMT are intolerant or develop resistance, especially in advanced stages of the disease, such as in the acute phase or blast crisis, restricting the effectiveness of its use [6,7,8,9,10]. To overcome resistance, new inhibitors based on the IMT structure have been developed, and second-generation TKIs (e.g., nilotinib, NLT; bosutinib, BST; and dasatinib, DST) have been introduced. Thus, there has been a significant expansion in drug therapy for this disease. However, the T315I mutation (resulting from the exchange of a threonine residue for an isoleucine residue at position 315 in the orthosteric site) is prevalent and is refractory to all available inhibitors [11,12]. This mutation occurs in a region within the catalytic site, known as the “gatekeeper”, which determines the size of the binding site for the endogenous substrate adenosine 5-triphosphate (ATP) [13]. In BCR-ABL1, this mutation reduces selectivity by preventing hydrogen bond interaction between inhibitors and the hydroxy group of threonine and an NH group present in the TKIs. Another issue related to this mutation is associated with the steric volume of isoleucine imposed on the catalytic site, preventing access to the adenine pocket by inhibitors but allowing access to ATP [6,13,14,15].

**Figure 1:**
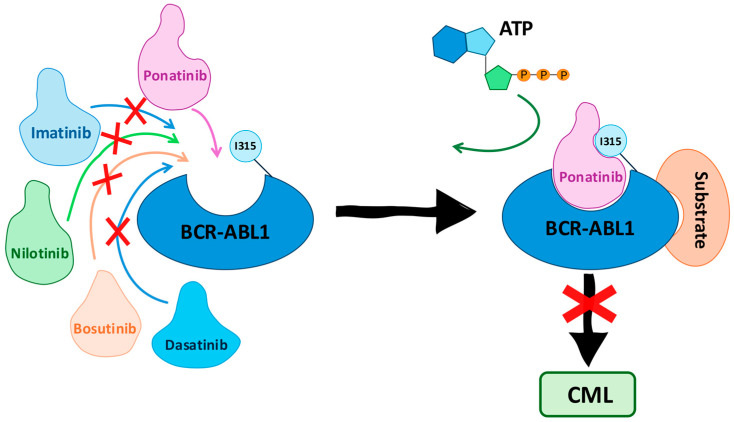
Mechanism of action of ponatinib in treating chronic myeloid leukemia (CML). Ponatinib effectively binds to the BCR-ABL1 protein, including the T315I mutant form, while other tyrosine kinase inhibitors are ineffective. Ponatinib occupies the ATP-binding site of BCR-ABL1, preventing ATP from binding and thus inhibiting the protein’s kinase activity. This blockade disrupts the signaling pathway that leads to CML, effectively halting the progression of the disease.

An essential segment of patients with CML, 15 to 26%, has the T315I mutation and does not respond satisfactorily to treatments with first- and second-generation inhibitors [16,17,18]. In 2012, Ariad Pharmaceuticals, a company focused on the development of oncology in the United States and currently part of the Takeda Pharmaceutical Company, developed ponatinib (PNT) as a multikinase TKI effective for treating patients with the T315I mutation (CML-T315I) [19]. The ponatinib unique structure allows it to bind to the ATP-binding pocket of the BCR-ABL1 kinase domain, including variants with the T315I mutation, which confers resistance to other tyrosine kinase inhibitors [20]. Upon binding, ponatinib forms critical hydrogen bonds and hydrophobic contacts within the kinase domain, effectively occupying the ATP-binding site. This occupation prevents ATP from binding and subsequently inhibits the phosphorylation of downstream substrates. By blocking the catalytic activity of BCR-ABL1, ponatinib disrupts the signaling cascades responsible for leukemic cell growth and survival (Figure 1) [21].

The efficacy of ponatinib extends to various BCR-ABL1 mutants due to its ability to maintain key binding interactions even in the presence of amino acid substitutions that typically confer resistance. Its approval by the Food and Drug Administration (FDA) occurred in the same year, establishing the third generation or third line of treatment for CML [22]. PNT was the result of research by Huang et al. on dual inhibitors capable of acting on both ABL1 and SRC (a kinase encoded by the Rous sarcoma virus proto-oncogene) because of high identity between their catalytic sites [23,24,25,26,27]. The overexpression of SRC family kinases has been associated with BCR-ABL1-induced leukemogenesis, especially in triggering B-cell acute lymphoblastic leukemia (ALL) and CML progression [28]. This simultaneous approach has already demonstrated its success for IMT, which acts both in CML therapy (BCR-ABL1 inhibitor) and in gastrointestinal stromal tumor (GIST) (c-Kit inhibitor) therapy, making it attractive to the pharmaceutical industry in Research and Development (R&D). Continuing with new TKI developments, DST was approved in 2006 for the treatment of adults in all stages of CML who are resistant or intolerant to IMT; DST is a potent inhibitor that acts on the SRC/ABL1 kinases [29].

Although PNT is the only ATP-competitive TKI available for the treatment of CML-T315I patients, it has been associated with significant and fatal adverse effects such as arterial, cardiovascular, cerebrovascular, and peripheral vascular occlusions. In a phase II study involving 449 patients, 26% experienced these adverse events [30]. Moreover, hypertension was observed in at least 25% of patients undergoing treatment, which led to five deaths [15]. These adverse cardiovascular events (ACVEs) prompted the temporary suspension of PNT in October 2013 [31]. In 2014, the sale of PNT was reauthorized due to the absence of another alternative; however, its labeling was updated to include revised and highlighted black box warnings about the risks of arterio-occlusive events (AOEs), heart failure, and hepatotoxicity [32,33,34,35,36]. The mechanisms of cardiovascular toxicity associated with the use of PNT are not yet completely understood. Additionally, some hypotheses suggest that off-target effects through interactions with specific kinases may contribute to the increased occurrence of ACVEs [15,33,37]. Even with several adverse effects, PNT is an important drug currently approved for the treatment of patients with CML who have the BCR-ABL1^T315I^ mutation.

Thus, it is fundamental to consider the PNT structure in the process of designing a new generation of TKIs. The aim of this review is to compile, in a chronological manner, PNT development, including data about its molecular target in CML therapy, computational methods, and experimental evaluation, to provide a solid and comprehensive foundation for future developments. This approach not only contextualizes the current state of knowledge about PNT but also sheds light on potential directions for future studies that may explore new therapeutic applications and pursue the ongoing optimization of this important inhibitor.

### 1.1. AP23464: Ponatinib Precursor

Blocking kinase-regulatory functions in the propagation and amplification of signals through ATP is one of the therapeutic strategies used in chemotherapy, also known as targeted therapy, to inhibit tumor development [38]. Half of the kinase enzymes, including tyrosine kinases (TKs) and serine/threonine kinases (STKs), are located in the cytoplasm [39]. TK exhibits structural similarity and utilizes the well-known “SRC module”, which comprises the SH3 and SH2 domains along with the kinase domain itself [40]. Notably, SRC and ABL1 share significant structural and sequence similarities in their binding sites [41,42]. Consequently, ATP-competitive inhibitors designed to target c-SRC are also effective ABL1 inhibitors [43].

The c-SRC enzyme is often overly active in solid tumors, and the homologous enzyme BCR-ABL1 is the primary molecular target for treating CML. c-SRC enzymes also play a vital role in hematological cancers such as CML and acute lymphoblastic leukemia (ALL) [43,44].

**Figure 2:**
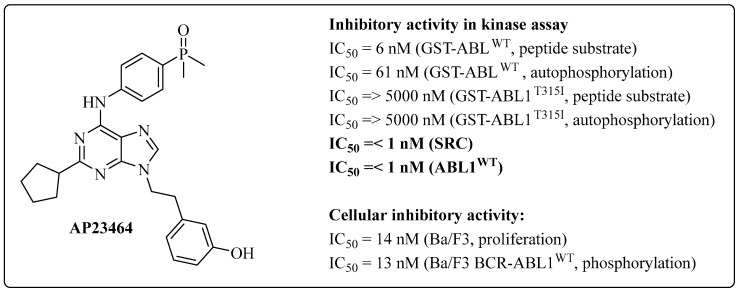
AP23464 chemical structure and activity data [47].

The development of dual inhibitors of the SRC/BCR-ABL1 kinases began in 1994 and 1995 when research groups investigated both targets, as described by Rickles et al. and Weng et al., respectively [45,46]. Since this initial study, researchers have focused their efforts on developing several series of dual inhibitors for the SRC and ABL1 kinases [23,42,47,48,49,50]. In 2004, O’Hare et al. developed an ATP-competitive prototype, AP23464 (3-[2-(2-cyclopentyl-6-{[4-(dimethylphosphoryl)phenyl]amino}-9*H*-purin-9-yl)ethyl]phenol), which has an IC_50_ lower than 1 nM for both kinases (ABL1 and c-SRC); this was the result of a screening study on synthetic libraries focusing on trisubstituted purine analogs that followed structural optimizations (Figure 2) [47].

AP23464 overcame a series of mutations that interfered with IMT therapy. This group concluded that AP23464 binds to the active conformation of the enzyme, unlike IMT, which prefers binding to the inactive conformation [51]. Thus, they proposed that AP23464 could complement treatment with IMT in cases of resistance [47].

AP23464 also inhibited phosphorylation in Ba/F3 cells transduced with BCR-ABL1^WT^ (wild type), with an IC_50_ value of 13 nM. Unmodified Ba/F3 cells were used as a control group to evaluate the inhibitory effect, with an IC_50_ of 14 nM (Figure 2) [23,47].

O’Hare et al. investigated the ability of AP23464 to inhibit ABL1 kinase activity in a cell-free system. Using GST-ABL1 fusion proteins, which combine the ABL1 kinase domain with glutathione S transferase (GST), the results confirmed the effectiveness of AP23464 in inhibiting ABL1 activity. The IC_50_ values were 61 nM for the autophosphorylation of GST-ABL1 and 6 nM for the peptide substrate of purified GST-ABL1 (Figure 2), demonstrating that AP23464 eliminates the tyrosine phosphorylation on BCR-ABL, blocks cell cycle progression, and promotes apoptosis in BCR-ABL-expressing cells. Additionally, biochemical assays with a purified GST-ABL kinase domain confirmed that AP23464 directly inhibits ABL activity [47].

Additionally, the prototype has demonstrated inhibitory efficacy at low concentrations (nanomolar) against mutants in the kinase domain of ABL1 that are resistant to IMT. These include mutants in the P-loop nucleotide-binding loop region, such as Q252H (IC_50_ = 12 nM), Y253F (IC_50_ = 54 and 13 nM), and E255K (IC_50_ = 110 nM and 14 nM); mutants in the C-terminal region, such as M351T (IC_50_ = 31 nM and 8 nM); and mutants in the activation loop region (*A*-loop), such as H396P (IC_50_ = 54 nM and 11 nM) [41,51,52]. AP23464 was ineffective against the mutant in the gatekeeper residue T315I (IC_50_ > 5000 nM for autophosphorylation and peptide substrate phosphorylation by ABL1) [47].

The following year, studies focused on mutations in the Kit kinase activation loop [48]. In 2006, Dalgarno et al. conducted molecular modeling studies with dual inhibitors and the SRC kinase [53], whereas Azam et al. expanded the panel of mutation variants related to IMT and tested AP23464 against these 58 variants [42].

**Figure 3:**
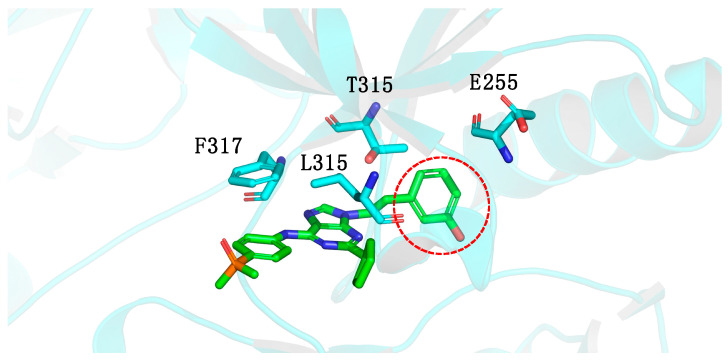
Three-dimensional representation of the lowest energy pose of AP23464 in a complex with the ABL1^T315I^ kinase. The dotted circle highlights the phenolic ring connected to the *N*-9 nitrogen atom of AP23464, which is positioned toward a deep hydrophobic cavity in the active site of the ABL1 kinase, and access to this cavity is limited by the T315I mutation [42].

Molecular modeling studies of AP23464 indicated that the phenolic ring linked to the *N*-9 nitrogen atom was directed to a deep hydrophobic cavity in the active site of the ABL1 kinase. However, access to this cavity was restricted by the T315I mutation (Figure 3) [42].

**Figure 4:**
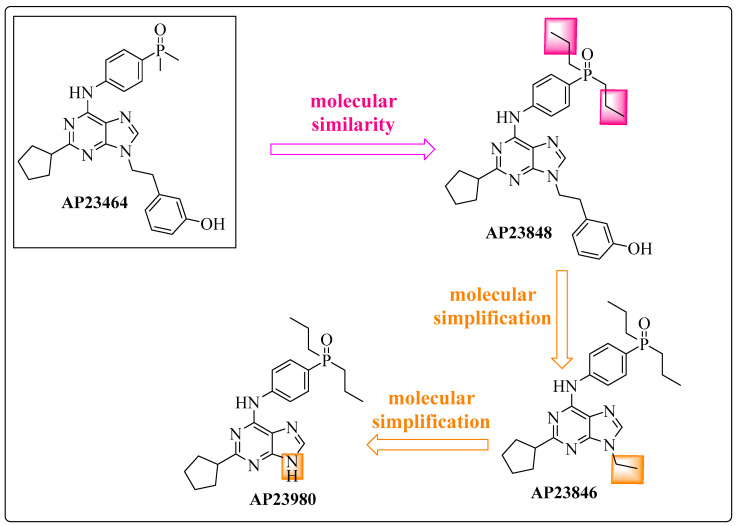
Chemical structure of AP23464 and its derivatives AP23848, AP23846, and AP23980, showing the structural modification process based on medicinal chemistry tools [42,47].

Based on the results obtained by mutagenesis and molecular modeling studies, Azam et al. developed derivatives of AP23464 to inhibit ABL1 containing the T315I mutation [42]. After specific structural changes, they obtained AP23848 (3-[2-(2-cyclopentyl-6-{[4-(dipropylphosphoryl)phenyl]amino}-9*H*-purin-9-yl)ethyl]phenol) using molecular similarity tools and then AP23980 (2-cyclopentyl-*N*-[4-(dipropylphosphoryl)phenyl]-9-ethyl-9*H*-purin-6-amine) and AP23846 (2-cyclopentyl-*N*-[4-(dipropylphosphoryl)phenyl)-9*H*-purin-6-amine) via molecular simplifications (Figure 4). The focus was the *N*-9 position of the purine ring because functionality at this position can interact with the T315I site [42].

In this series of compounds, the 4-dimethylphosphoryl fragment of AP23464 was replaced by the 4-dipropylphosphoryl of AP23848 (Figure 4, in pink) through a high-similarity strategy (increase or decrease in the number of methylene groups, in this case, increase) [54]. This modification increased the lipophilic character of the prototype, resulting in the derivative AP23848, which was significantly more active than its precursor with an IC_50_ of 5.1 nM against ABL1^T315I^ (Figure 4) (Table 1) [42]. This increase in the selective inhibition activity was accompanied by an increase in its partition coefficient (clogP = 5.36) compared with that of the precursor AP23464 (clogP = 3.99) [55] (Table 1), and this increased clogP may create problems with oral absorption and toxicity.

The *N*-9 position of the purine ring was subsequently chosen as the point of molecular modification in the pharmacophoric group, resulting in the derivatives AP23846 and AP23980. The first had the phenolic ring removed (Figure 4, in orange) and demonstrated an IC_50_ of 6.4 nM against ABL1^T315I^, similar to AP23848 [42]. In terms of lipophilicity, AP23846 presented a clogP of 4.62, 25% lower than that of the analog AP23848 (clogP = 5.36) [55] (Figure 4) (Table 1). These results suggest that the AP23846 analog may exhibit more favorable pharmacokinetic properties compared with AP23848. For the AP23980 analog, the ethyl group present in AP23846 (IC_50_ = 6.4 nM) was removed (Figure 4, in orange), resulting in a loss of inhibitory potency of almost 50 times (IC_50_ = 297 nM) [42] and increased lipophilicity (clogP = 4.62) (Table 1).

**Figure 5:**
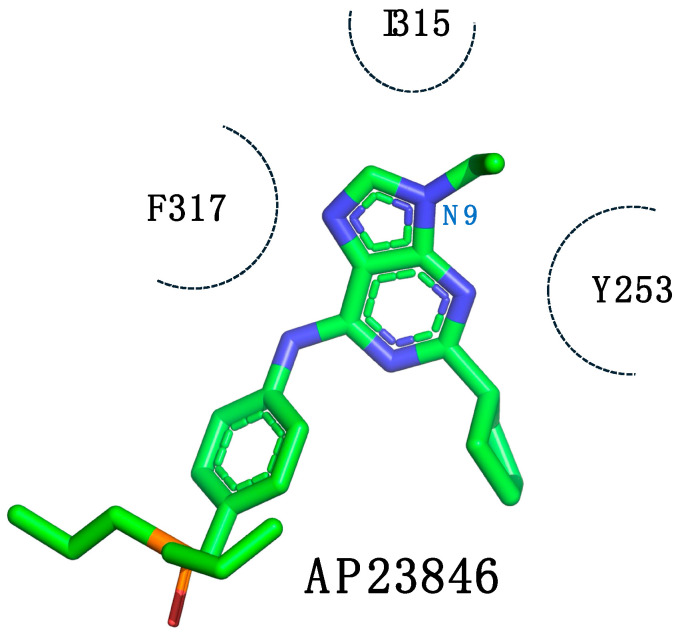
Three-dimensional representation of AP23846 docked to the active site of the ABL1 kinase [42].

These results suggest that the withdrawal of the bulky phenol group and the addition of alkyl chains to the phosphoryl group allowed for increased selectivity against the T315I mutation (Figure 5).

Unfortunately, AP23846 is not considered a suitable drug candidate because of its nonspecific cytotoxicity. Its ability to inhibit the proliferation of parental BaF3 cells suggests the possibility of off-target effects [42].

### 1.2. Conformational Restriction of the Spacer as a Medicinal Chemical Tool: Insertion of the Ethylene Group

In 2008, Wang et al. summarized the structural optimization of the active analog AP23464 with exciting results in the dual inhibition of SRC and ABL1 [49]. Through molecular docking studies, instead of the ethyl spacer linked to the *N*-9 position of the purine nucleus, the presence of an ethylene group (diastereoisomer *E*) as a spacer could guide a hydrophobic group to the region of selectivity of these kinases [56]. The goal was to allow the phenol group to reach this region; this hydrophobic pocket is close to the ATP binding site, as observed by the 2-chloro-6-methylphenyl moiety of the TKI DST in c-SRC (PDB: 3G5D) [57] and ABL1 (PDB: 2GQG) [58].

**Figure 6:**
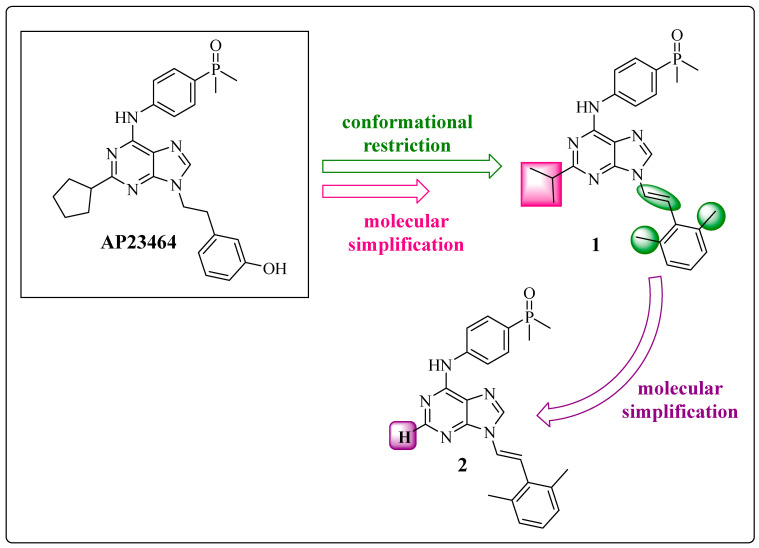
Chemical structures of **1** and **2** (purines *N*9-arentenyl) [49].

Among the derivatives synthesized, compounds **1** 9-[(1*E*)-2-(2,6-dimethylphenyl)ethenyl]-*N*-[4-(dimethylphosphoryl)phenyl]-2-(propan-2-yl)-9*H*-purin-6-amine and **2** 9-[(1*E*)-2-(2,6-dimethylphenyl)ethenyl]-*N*-[4-(dimethylphosphoryl)phenyl]-9*H*-purin-6-amine presented greater inhibitory potency against ABL1^wt^ (IC_50_ of 15.8 nM and 3.58 nM, respectively) and favorable pharmacokinetic properties (Figure 6) (Table 2) [49]. This work suggests that 1,3-dimethylbenzene is an aromatic system that presents an ortho effect provided by its two methyl groups, reducing their conformational freedom and acting as a metabolic protector of the double bond of the spacer (Figure 6, in green).

The cyclopentane portion of compound **1** was replaced by an isopropyl group through molecular simplification, reducing the structural complexity but maintaining the pharmacophoric groups [59] (Figure 6, in pink). This change increased the inhibitory potency against wild-type ABL1 and lipophilicity by 17% (clogP = 4.67) compared with the precursor (Table 2). In contrast, compound **2** was prepared by a molecular simplification of compound **1**, with the removal of the isopropyl subunit (Figure 6, in purple). Compound 2 maintained the dual activity, which suggests that groups in this position are auxophores. This change reduced the lipophilicity (clogP = 3.68) compared with that of compound **1** (clogP = 4.67) and AP238464 (clogP = 3.99) (Table 2) [49]. As observed in derivative **2**, the cyclopentane group linked to the purine core at position C-2 was removed without disrupting activity and as a requirement due to steric factors and possibly unfavorable interactions with residues of the glycine-rich loop (such as Leu248 and Tyr253) [23].

Only compound **2** was subjected to enzymatic assays including testing against a panel of 35 kinases. Compound **2** did not exhibit inhibitory activity toward ABL^T315I^ [49].

### 1.3. The Insertion of Arylamines: Targeting the Deep Hydrophobic Pocket of the Enzyme

Until 2009, prototype design was guided toward the active conformation because of the significant similarity in this state between ABL and SRC and the desire for dual activity [29,60]. In the same year, Huang et al. classified the inhibitors according to the state of the enzymes as active or inactive [23]. The active conformation depends on the favorable position of the catalytic triad of aspartate (D), phenylalanine (F), and glycine (G) located in the activation loop (A-loop) and enables the phosphorylation of substrates through the transfer of gamma-phosphate from ATP in the catalytic site of the enzyme. In this state, known as DFG-in, there may be direct competition between the inhibitors and ATP for the catalytic site. Nevertheless, in the inactive or latent conformation, phosphorylation does not occur without ATP binding, also known as DFG-out, and in this case, the inhibitor acts though a conformational selection with subsequent induced fit, inhibiting the enzyme in its latent state [23].

**Figure 7:**
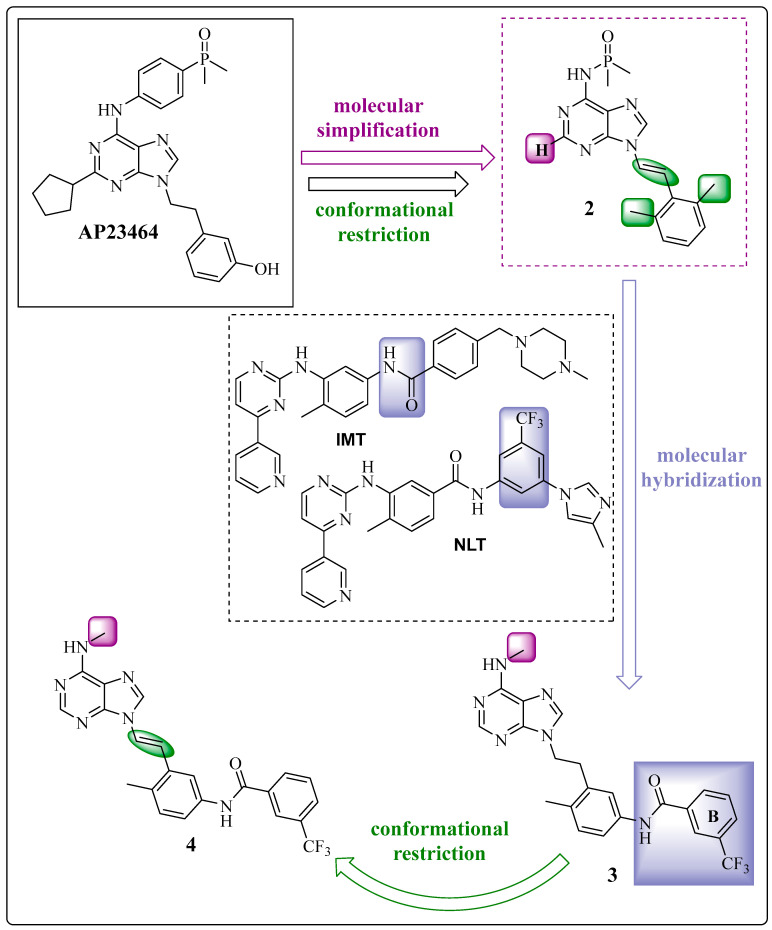
Chemical structures of compounds **3** and **4**, showing the structural modification process based on medicinal chemistry methods [23,49].

From the prototype AP23464 and derivative **2**, which were previously developed by Wang et al. via molecular docking simulations, compounds **3** *N*-(4-methyl-3-{2-[6-(methylamino)-9*H*-purin-9-yl]ethenyl}phenyl)-3-(trifluoromethyl)benzamide and 4 *N*-(4-methyl-3-{(1*E*)-2-[6-(methylamino)-9*H*-purin-9-yl]ethyl}phenyl)-3-(trifluoromethyl)benzamide were prepared (Figure 7) [49]. First, the dimethylphenylphosphine oxide group at the C-4 position was removed from the exocyclic nitrogen of the purine nucleus, which was replaced by a methyl group in product **3** (Figure 7, in purple) through a molecular simplification. However, the main change in the proposed derivatives **3** and **4** was based on molecular hybridization between compound **2** and the amide and 3-trifuoromethylbenzene fragments from the structures of IMT and NLT, respectively [61] (Figure 7, in lilac), making the prototype more accurate. This inclusion aimed to reach the deepest cleft in the enzyme provided by the DFG-out conformation. Another important change was the maintenance of the methyl group (Figure 7, in blue), which was essential for increasing the selectivity against BCR-ABL1, as observed for IMT and NLT [62,63,64].

The main difference between derivatives **3** and **4** is the conformational restriction. Derivative **4**, which presents conformational restrictions, is nearly four times as potent as derivative **3** (IC_50_ = 25 nM and IC_50_ = 96 nM, respectively) (Table 3) [23]. Despite the high similarity of the molecular targets, there is a gain in the selectivity of these compounds for ABL1. There was an increase in lipophilicity relative to its precursors but no significant difference between **3** (clogP = 4.10) and **4** (clogP = 4.13) (Table 3) [55].

**Figure 8:**
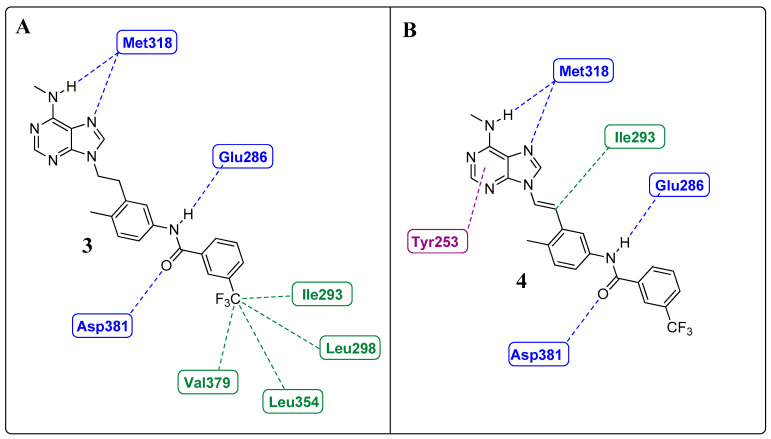
(**A**). Two-dimensional interaction map between **3** and ABL1 residues (DFG-out conformation). (**B**). Two-dimensional interaction map between residues **4** and ABL1 (DFG-out conformation). The hydrogen bonds and van der Waals interactions are represented by blue and green dotted lines, respectively; putative pi-stacking interactions are represented by purple dotted lines [23].

Huang et al. and Sherman et al. observed, from crystallographic structures, essential hydrogen bonds between the amide group of the inhibitors and the DFG-out conformation of ABL1 that allowed access to the deep cleft of the enzyme [23,65]. Therefore, they conducted molecular docking studies to investigate the interactions of those derivatives with the ABL1 kinase in its inactive conformation (DFG-out), the same as that to which IMT binds (PDB: 1IEP). Compound **3** established four hydrogen bond interactions with the molecular target. Two of these interactions occurred in the “hinge” region of the kinase (ATP adenine recognition region), one through the *N*-7 nitrogen of the purine nucleus and the other through the NH of the methylamino group on the C-6 carbon, both of which interact with the NH residue (Met318). The amide group established two more hydrogen interactions: one with the side chain of Glu286 in α-helix C and another with Asp381 in the DFG moiety. The trifluoromethylphenyl subunit occupies a hydrophobic site that is only available in the DFG-out conformation [66], providing van der Waals-type interactions with residues Ile293, Leu298, Leu354, and Val379 (Figure 8A) [23].

The authors reported four hydrogen bond interactions between compound **4** and its molecular target, ABL1 (Asp381, Glu286, and Met318), as observed for compound **3** (Figure 8B). Furthermore, they highlighted the existence of a putative pi-stacking interaction between the Tyr253 residue and the purine nucleus and a hypothetical interaction between the ethenyl group and Ile315 (mutated gatekeeper) (as illustrated in Figure 8B) or Thr315 (wild-type gatekeeper). The latter was described as an unconventional hydrogen bond type with the proton of the olefinic bond [67]. Notably, these derivatives occupy the same region of the orthosteric site, with interactions like those of IMT and NLT [23].

**Figure 9:**
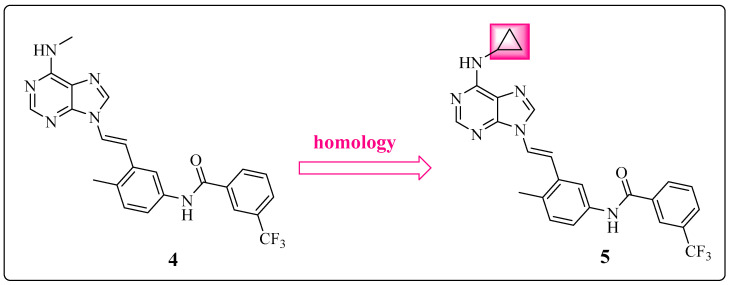
Chemical structures of compound **4** and its derivative **5**, showing their respective in vitro activities [23].

The in vitro biological evaluation of the compounds included determining the inhibitory activity of the SRC and ABL1 kinases (Figure 7 and Figure 9). However, with a focus on suppressing the activity of the BCR-ABL1 kinase, they used K562 cells (a human CML cell line that expresses the wild-type variant of the BCR-ABL1 enzyme), Ba/F3 BCR-ABL1^WT^ cells, and unmodified Ba/F3 cells [23]. Compound **4** presented IC_50_ values of 52 nM and 25 nM against the SRC and ABL1 kinases, respectively, and these results are in accord with the in silico results (Table 4). In the evaluation of K562 and Ba/F3 BCR-ABL^WT^ cells, **4** also showed potency, with IC_50_ values of 67 nM and 47 nM, respectively (Table 4). Furthermore, there was no effect on the parental control group (Ba/F3) at concentrations of up to 10 μM, highlighting its selectivity, as shown in Figure 9 [23].

However, compound **4** was quickly eliminated after oral administration in rats. This finding is likely related to the methylamino group at the C-6 carbon of the purine ring [23], because the *N*-phenyl fragment of 3-trifluoromethyl-benzamide, present in other inhibitors, is associated with good oral bioavailability [63,64].

Based on these results, the methylamino group seems to be a consequence and not a cause of low bioavailability. Among the enzymes of the CYP450 superfamily, CYP2D6 is particularly responsible for the *N*-dealkylation of xenobiotics, with a direct consequence on the conjugation of the second phase of metabolism and subsequent excretion [68]. The data simulated via SmartCyp [69] suggested that the methyl group of derivative **4** is more susceptible to metabolism by this CYP compared with the cyclopropyl group used at the same position in derivative **5**. This fact is consistent with the change proposed by Huang et al. through the application of high similarity (Figure 9) [23].

Compound **5** *N*-{3-[(1*E*)-2-[6-(cyclopropylamino)-9*H*-purin-9-yl]ethenyl}phenyl)-3-(trifluoromethyl)benzamide exhibited a good pharmacokinetic profile. It remained largely unchanged in the systemic circulation, with a high area under the curve (AUC) of 32151 and a robust oral bioavailability of 40%. Potency toward the target kinase (ABL1^WT^ = 74 nM) and cellular activity (K562 = 95 nM; Ba/F3 BCR-ABL1^WT^ = 57 nM) were retained. Furthermore, compound **5** (cLogP = 4.57) exhibited increased lipophilicity compared to its precursor (cLogP = 4.13) without compromising pharmacokinetic parameters (Table 4) [23,55].

#### Learning from IMT and NLT: The Importance of the Position of the Amide Group

**Figure 10:**
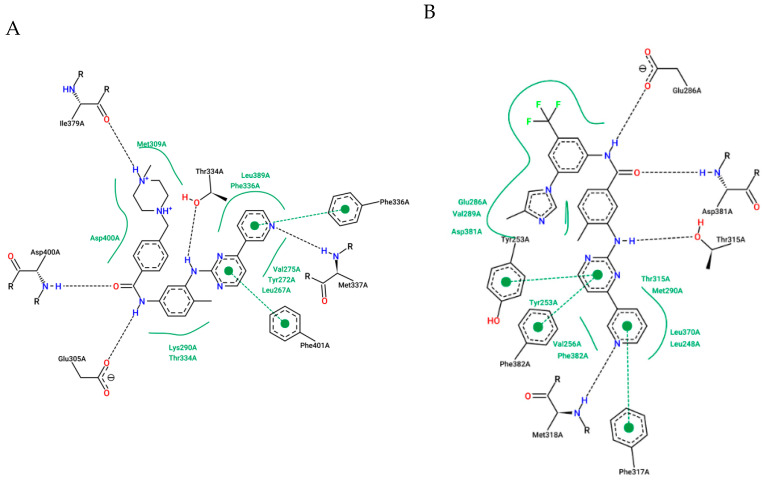
(**A**) Two-dimensional interaction map between the IMT and ABL1 residues. (**B**) Two-dimensional interaction map between NLT and ABL1 residues.

The crystal structure of IMT cocrystallized in the enzyme BCR-ABL1^WT^ (PDB code: 2PYY) [70] revealed crucial interactions through hydrogen bonds to Glu305A (286), Asp400A (381), and Met337A (318A) (Figure 10A).

On this basis, NLT (AMN-107) was developed to be more effective and selective than IMT, bypassing mutations that influence the treatment of some cases [71,72]. Consequently, the main modifications relative to IMT included the inversion of the amide group (a retroamide), the insertion in the meta positions of aromatic ring B of the trifluoromethyl group, and the addition of 1-methylimidazole (Figure 10B) (PDB code: 3CS9). These modifications preserved the same hydrogen bonds as IMT with the residues Glu286A, Asp381A, and Met318A of the enzyme. The insertion of the trifluoromethyl group and 1-methylimidazole expanded the hydrophobic contacts favorably, as did an additional interaction between the carbonyl of the Asp381A group and the fluorine atom of the trifluoromethyl group [73]; this resulted in a tenfold increase in the potency of NLT (IC_50_ = 43 ± 15 nM) compared with that of IMT (IC_50_ = 470 ± 59 nM) against autophosphorylation in K562 cells [74].

**Figure 11:**
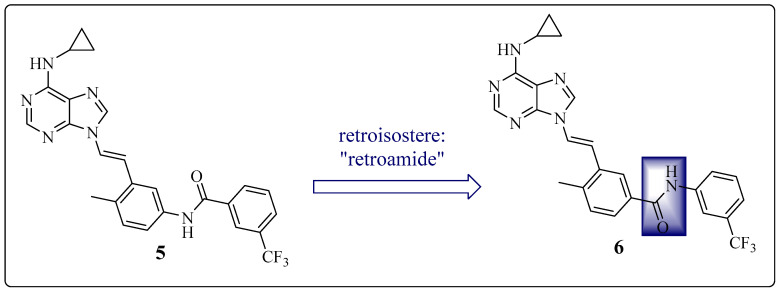
Chemical structures of **5** and its derivative **6**, showing their respective inhibitory activities against SRC and wild-type ABL 1 [23].

Therefore, Huang et al. investigated the influence of the amide position on the *N*-(p-tolyl)-3-(trifluoromethyl)benzamide subunit. Compound **5** presents an amide group, as in IMT, whereas compound **6** 3-[(1*E*)-2-[6-(cyclopropylamino)-9*H*-purin-9-yl]ethenyl]-4-methyl-*N*-{3-(trifluoromethyl)phenyl}benzamide has a retroamide group, similar to NLT. This change resulted in a threefold increase in the potency of the new derivative (**6**) compared with that of precursor **5** (Figure 11) (Table 5) [23].

### 1.4. Focus on the Selective Hydrophobic Pocket in the Deep Region of the Kinase: The Terminal Aromatic Ring of Compounds

**Figure 12:**
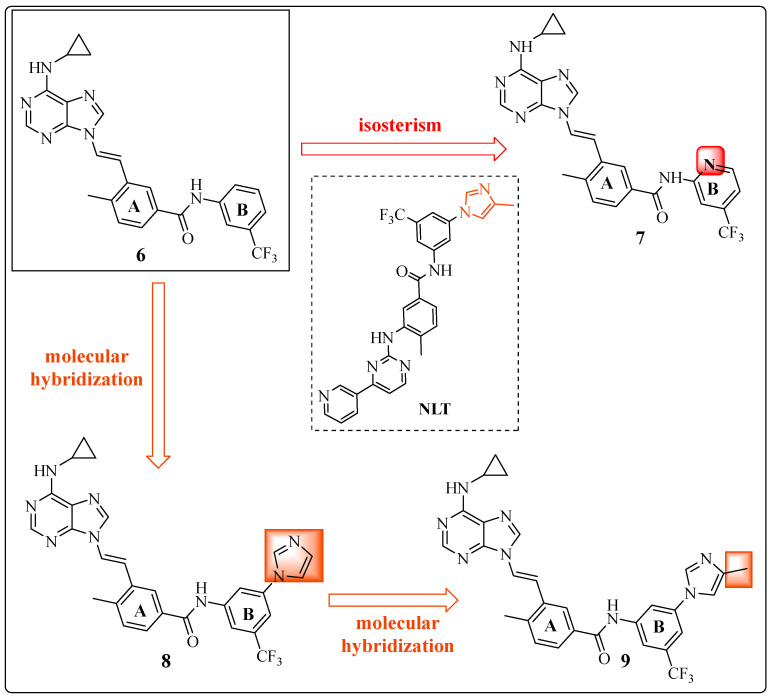
Chemical structures of derivatives **7**, **8**, and **9**, showing the medicinal chemistry methods applied in each step [23].

After defining the conformational restriction as crucial for the activity of the developed inhibitors, structural optimizations were proposed to target the hydrophobic cavity in the deep region of the kinase, arising from the movement of the DFG triad. On the basis of structure–activity relationships (SARs), the B ring (Figure 12) is a target for structural modifications, motivated by the structural difference between the NLT and IMT inhibitors. A new series of derivatives of **6** was produced, resulting in compounds **7** 3-[(1*E*)-2-[6-(cyclopropylamino)-9*H*-purin-9-yl]ethenyl]-4-methyl-*N*-[4-(trifluoromethyl)pyridin-2-yl]benzamide, **8** 3-[(1*E*)-2-[6-(cyclopropylamino)-9*H*-purin-9-yl]ethenyl]-*N*-[3-(1H-imidazole-1-yl)-5-(trifluoromethyl)phenyl]-4-methyl]benzamide, and **9** 3-[(1*E*)-2-[6-(cyclopropyl amino)-9*H*-purin-9-yl]ethenyl]-4-methyl-*N*-[3-(4-(methyl-1*H*-imidazol-1-yl)-5-(trifluoromethyl)phenyl]benzamide (Figure 12) [23].

Compound **7** was prepared via isosterism tools (precise changes in the precursor with the maintenance of complexity and functional groups) [75] with the replacement of the aromatic ring of compound **6** with a heteroaromatic ring (pyridyl moiety). The maintenance of dual activity (IC_50_ = 7 nM (c-SRC) and IC_50_ = 20 nM (ABL1)) [23] was observed, but there was a 20% reduction in lipophilicity (cLogP = 3.92 to cLogP = 3.12) [55] (Figure 12) (Table 6), which may favor the balance between aqueous water solubility and lipid solubility.

Compounds **8** and **9** were rationalized based on the insertion of groups present in the structure of NLT in the ABL1 region, using the molecular hybridization tool between NLT and compound **6**. In compound **8**, the imidazole ring present in NLT was inserted. In addition, in compound **9**, the 4-methyl-1*H*-imidazole group was inserted (both in dark orange, Figure 12).

The screening tests were conducted using those derivatives in the ABL1^T315I^ kinase assay and, subsequently, in Ba/F3 cells transfected with BCR-ABL1^T315I^. Compounds **8** and **9**, which contain the imidazole moiety at the meta position of ring B, moderately inhibited the ABL1^T315I^ kinase (IC_50_ = 542 nM and IC_50_ = 478 nM, respectively). Furthermore, these compounds have been demonstrated to inhibit the proliferation of transfected Ba/F3 cells (BCR-ABL^T315I^), with IC_50_ values of 298 nM and 422 nM, respectively (Figure 12) (Table 6) [23].

**Figure 13:**
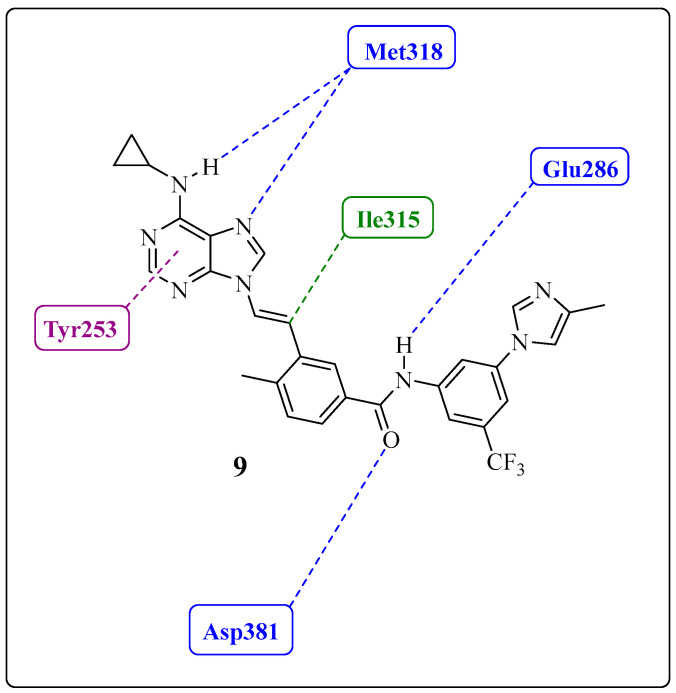
Two-dimensional interaction map between compound **9** and the ABL1 residue. Hydrogen bonds and putative pi-stacking interactions are represented by blue and purple dotted lines, respectively. Additionally, hydrophobic interactions are represented by the green dotted line.

Based on crystallographic data of compound **9** cocrystallized with the mutant enzyme (ABL1^T315I^) and molecular modeling studies, the 4-methylimidazole group interacts with the residues Glu228, Glu282, Val289, Asp381, and Phe359 in the hydrophobic pocket of the enzyme [23]. The researchers concluded that these interactions contribute effectively through these additional hydrophobic contacts with the mutant protein kinase. Some interactions of the complex between compound **9** and ABL1 can be observed in Figure 13.

Huang et al. evaluated the antiproliferative activity of these compounds in a comprehensive panel of Ba/F3 cells expressing clinically relevant mutations in the BCR-ABL1 kinase, including T315I, L248R, G250E, Q252H, Y253H, and E255K. Compound **9** was more active against the T315I mutation and presented IC_50_ values of 64, 63, 42, 44, and 24 nM in Ba/F3 cells expressing the other mutants, respectively [23]. This derivative proved to be more potent than NLT against these mutations.

In an assay that evaluated the effects on autophosphorylation and proliferation in Ba/F3 cells transfected to express native forms of the BCR-ABL1 enzyme and resistant to IMT, NLT showed autophosphorylation IC_50_ values of 92, 117, 260, 153, and >10,000 nM for the G250E, Q252H, Y253H, E255K, and T315I mutations, respectively [74]. Despite showing moderate efficacy against the T315I mutation in ABL1, derivative **9** (also known as AP24163) is promising because of its refractory behavior in tests with first- and second-generation inhibitors available for treatment [23].

**Figure 14:**
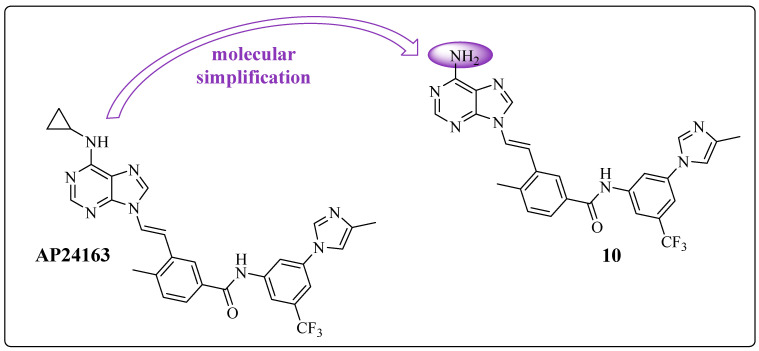
Chemical structures and results of in vitro tests of prototype **9** (AP24163) and its derivative **10** [23].

From the active **9** (AP24163), Zhou et al. proposed a new modification focusing on the hinge region, the adenine recognition region of ATP, resulting in derivative **10** (Figure 14) [50]. They applied a molecular simplification to remove the cyclopropyl moiety from the exocyclic nitrogen of the purine ring.

Both compounds, AP24163 and **10**, exhibited moderate activity against BCR-ABL1^T315I^ in cellular assays, with IC_50_ values of 478 and 386 nM, respectively. Furthermore, these compounds also inhibited native ABL1, as demonstrated in Table 7 [50]. Derivative **10** showed better potency toward the mutant enzyme and lower lipophilicity (cLogP = 3.41) (Table 7) [55].

### 1.5. Large Structural Change in the T315I Mutation: The Introduction of Acetylene as a Spacer

**Figure 15:**
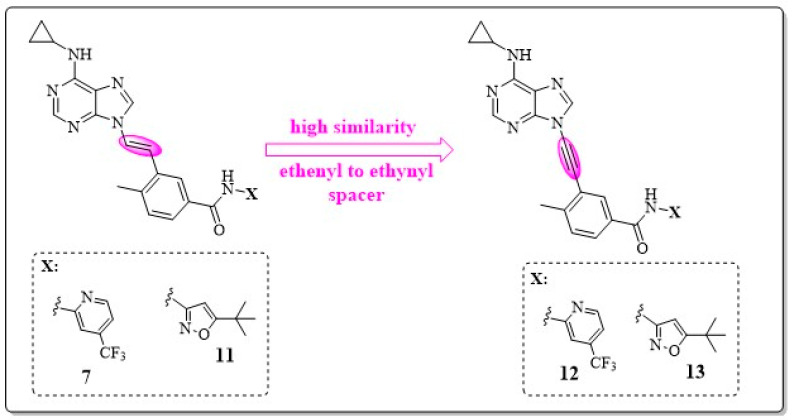
Chemical structures of compounds containing the ethenyl moiety (**7** and **11**) and their homologues with ethynyl moieties (**12** and **13**) [17].

After promising results were obtained with analog **9** (AP24163), researchers focused on the use of other spacers with conformational restrictions. Two sets of compounds, ethylenic and acetylenic derivatives, were developed to evaluate activity toward ABL1^T315I^. The acetylenic derivatives were designed from ethylenic compound **7** and derivative **11** through a highly similar process (Figure 15, in pink). The compounds **12** 3-{2-[6-(cyclopropylamino)-9*H*-purin-9-yl]ethynyl}-4-methyl-*N*-[4-(trifluoromethyl)pyridin-2-yl]benzamide and **13** *N*-(5-tert-butyl-4,5-dihydro-1,2-oxazol-3-yl)-3-{2-[6-(cyclopropylamino)-9*H*-purin-9-yl]ethynyl}-4-methylbenzamide showed greater potency in enzymatic and antiproliferation assays in cells compared with their respective ethylenic precursors.

The researchers found that changing the functional group is crucial for improving activity with small variation in lipophilicity, as shown in Figure 15 and Table 8 [17,55].

**Figure 16:**
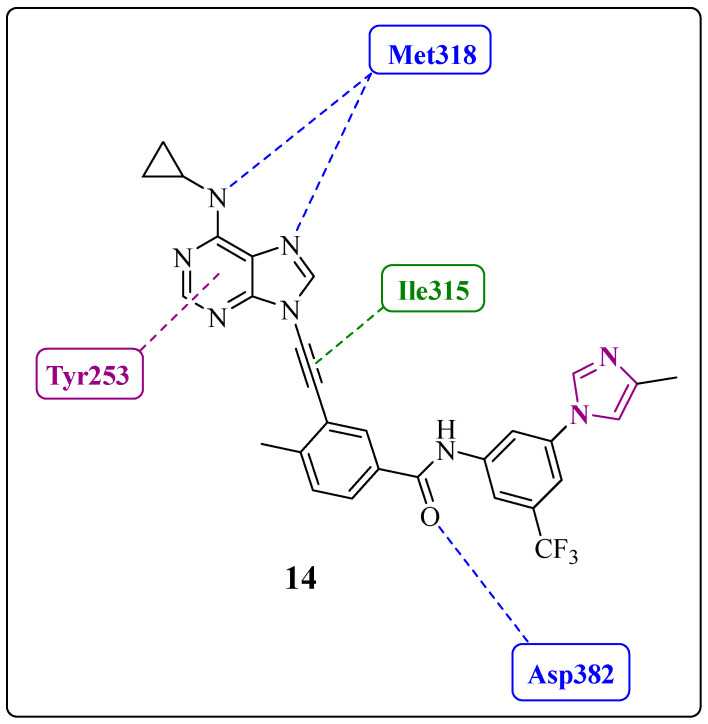
Two-dimensional interaction map between **14** and ABL1 residues (DFG-out conformation). The hydrogen bonds and van der Waals interactions are represented by blue and green dotted lines, respectively; putative pi-stacking interactions are represented by purple dotted lines [17].

The virtual nonsynthesized compound **14** 3-{2-[6-(cyclopropylamino)-9*H*-purin-9-yl]ethynyl}-4-methyl-*N*-[3-(4-methyl-1*H*-imidazol-1-yl)-5-(trifluoromethyl)phenyl]benzamide was used only in the molecular docking study, in which it showed a better fit in the active site of the enzyme (Figure 16) [17]. The main interactions of the ligand–protein complex include hydrogen bond interactions (Met318 and Asp382), hydrophobic contacts, and van der Waals interactions (Ile315), in addition to a potential pi-stacking interaction (Tyr253) (Figure 16) [17].

However, acetylenic and ethylenic derivatives showed pharmacokinetic issues when administered orally to rats, including low exposure and a short half-life [17], indicating the need to optimize their properties.

### 1.6. Final Adjustments: Focus on the Hinge Region and ADMET Properties

**Figure 17:**
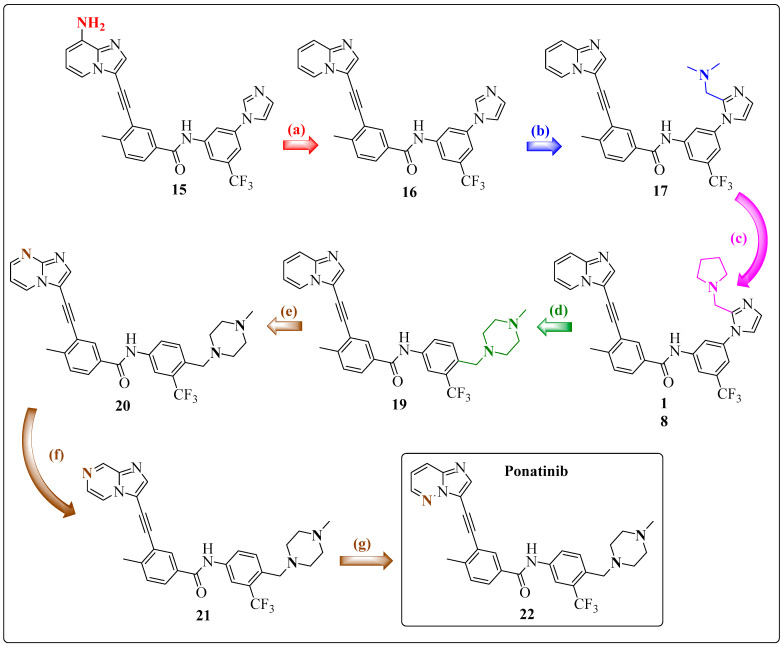
Structural optimizations of compounds were performed prior to the discovery of PNT, highlighting the structural modifications with different colors and displaying the biological results [17]. (**a**) Molecular simplification (red); (**b**) insertion of the *N,N*-dimethylethanamine group (blue); (**c**) high similarity (pink); (**d**) molecular simplification (green); (**e**–**g**) regioisomers (brown).

Researchers have taken a different approach to solve the shortcomings of 9-arenylethynyl purine-based inhibitors. Instead of using N-C≡C-C structures, which are present in the purine series, they use C-C≡C-C structures, which are more chemically and pharmacologically stable. The 8-amino-imidazolo[1,2-*a*]pyridine ring system was chosen because it was predicted that the C-8 (NH_2_) and *N*-1 positions of this system would maintain crucial hydrogen bond-type interactions in the region of the ABL1 kinase hinge. Among the designed compounds, the inhibitor **15** 3-(2-{8-aminoimidazo[1,2-*a*]pyridin-3-yl}ethynyl)-*N*-[3-(1*H*-imidazol-1-yl)-5-(trifluoromethyl)phenyl]-4-methylbenzamide was more effective against ABL^T315I^ compared with compounds **14**, **16**, and **17** while preserving native ABL1 activity; however, it exhibited low oral bioavailability in rats (Figure 17) (Table 9) [17].

Because of the unfavorable results with compound **15**, whether the problem was related to the exocyclic amino group at the C-8 position of the 8-amino-imidazolo[1,2-*a*]pyridine ring or to the aromatic-acetylene-aromatic system was investigated. Previously, a similar series indicated that the free amino or acetamide group at the position equivalent to the C-8 carbon often produced undesirable pharmacokinetic results. Thus, the analog **16** 3-(2-{imidazo[1,2-*a*]pyridin-3-yl}ethynyl)-*N*-[3-(1*H*-imidazol-1-yl)-5-(trifluoromethyl)phenyl]-4-methylbenzamide, which has no substitution at position C-8 (as represented in Figure 16), was synthesized and evaluated. Compound **16** exhibited satisfactory in vitro activity and, notably, demonstrated excellent oral exposure (AUC 0–24 h/dose = 5476 ng/h/mL/mg/kg) and good oral bioavailability (42%) (Table 9) when tested in rats [17].

**Figure 18:**
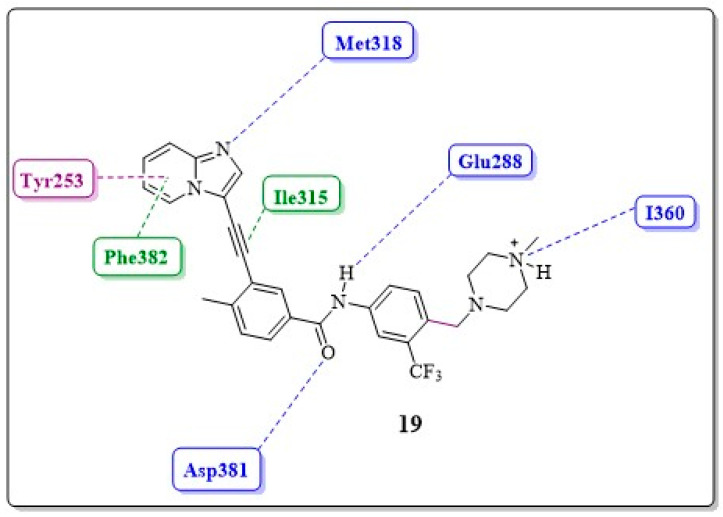
Two-dimensional interaction map between **19** and ABL1 residues (DFG-out conformation). The hydrogen bonds and van der Waals interactions are represented by blue and green dotted lines, respectively; putative pi-stacking interactions are represented by purple dotted lines [17].

Compound **16** has a favorable pharmacokinetic profile, but its cellular potency against ABL1^WT^ is lower than that of compound **15** (Table 9). Researchers have attempted to enhance its potency by introducing water-soluble amines into the imidazole ring, but this approach did not improve results (see **17** *N*-(3-{2-[(dimethylamino)methyl]-1*H*-imidazol-1-yl}-5-(trifluoromethyl)phenyl)-3-(2-{imidazo[1,2-*a*]pyridin-3-yl}ethynyl)-4-methylbenzamide and **18** 3-(2-imidazo[1,2-*a*] pyridin-3-yl}ethynyl)-4-methyl-*N*-(3-{2-[(pyrrolidin-1-yl)methyl]-1*H*-imidazol-1-yl}-5-(trifluoromethyl)phenyl)benzamide, Figure 17). Similarly, they designed a new inhibitor, **19** 3-(2-{imidazo[1,2-*a*]pyridin-3-yl}ethynyl)-4-methyl-*N*-{4-[(4-methylpiperazin-1-yl)methyl]-3-(trifluoromethyl)phenyl}benzamide, which has a methylpiperazine unit at the C-4 carbon of ring B, similar to IMT. This group is typically employed to improve the solubility of compounds. It is expected that the terminal nitrogen atom of piperazine would be protonated at physiological pH, and the subsequent quaternary salt would form a hydrogen bond with the carbonyl oxygen atom of residue Ile360 in the protein activation loop. These improved molecular interactions should increase the potency against native ABL1 and ABL1^T315I^ (Figure 18) [17].

Compared with compound **16**, compound **19** demonstrated a 2-fold increase in activity against BCR-ABL1^T315I^ (Figure 17) (Table 9) and was approximately 20 times more potent in inhibiting the growth of Ba/F3 cells expressing BCR-ABL^T315I^. Furthermore, compound **19** has shown a lower tendency to bind to plasma proteins compared with compound 16, had favorable oral absorption when administered to rats and mice, and was identified as the first orally active inhibitor of BCR-ABL1^T315I^ in a pilot efficacy experiment [17].

Researchers reported that by replacing carbon atoms with nitrogen in three regioisomers of pyridine rings, oral bioavailability was significantly reduced with each substitution. During the study, inhibitor **20** 3-(2-{imidazo[1,2-*a*]pyrimidin-3-yl}ethynyl)-4-methyl-*N*-{4-[(4-methylpiperazin-1-yl)methyl]-3-(trifluoromethyl)phenyl}benzamide with a nitrogen at the C-8 position as a hydrogen bond acceptor significantly reduced the potency (Table 9). However, the inhibitors **21** 3-(2-{imidazo[1,2-*a*]pyrazin-3-yl}ethynyl)-4-methyl-*N*-{4-[(4-methylpiperazin-1-yl)methyl]-3-(trifluoromethyl)phenyl}benzamide and **22** 3-(2-{imidazo[1,2-*a*]pyridazin-3-yl}ethynyl)-4-methyl-*N*-{4-[(4-methylpiperazin-1-yl)methyl]-3-(trifluoromethyl)phenyl}benzamide have demonstrated greater potency in both enzymatic and cell assays compared with **19**. Compound **22** has exhibited more promising preliminary pharmacokinetics compared with **21**, which led to a more detailed analysis of its SAR by Huang et al. Inhibitor **22** has demonstrated remarkable cellular potency in the nanomolar range against cell lines expressing both native BCR-ABL1 and BCR-ABL^T315I^, as illustrated in Table 9. It also prolonged survival in an aggressive mouse model of CML driven by the T315I mutation. In addition, it potently inhibited the proliferation of all clinically relevant ABL1 mutants in cell-based assays, making it the first pan-BCR-ABL1 inhibitor. Notably, it was assigned the code AP24534 and later named PNT [17].

It is important to highlight that the results of the cellular assays (ABL1^wt^ and ABL1^T315i^) and oral bioavailability (F%) of compounds **15**, **16**, **17**, **18**, **19**, **20**, **21**, and **22** are presented in Table 9.

### 1.7. Ponatinib (PNT)

Preclinical trials of PNT were initially described by O’Hare et al. in 2006 [76,77]. These in vitro studies used Ba/F3 cell lines that expressed native BCR-ABL1 or BCR-ABL1 with various mutations in the ABL1 kinase domain. Kinase specificity tests have demonstrated its efficacy in inhibiting both wild-type and mutant variants of the BCR-ABL1 kinase, including BCR-ABL^T315I^, with IC_50_ values of 0.37 and 2 nM, respectively [22,76]. This study also investigated the efficacy of PNT at various concentrations (0–1000 nM) against mononuclear cells from patients with leukemia in blast crisis (an advanced stage of the disease) driven by BCR-ABL1 or BCR-ABL1^T315I^, and these results were compared with those from healthy individuals. Cell proliferation assays revealed that PNT significantly reduced the number of viable primary CML cells, with IC_50_ values approximately 500 times lower than those observed in normal cells, confirming its potent comprehensive inhibitory effect toward BCR-ABL1 [22,76].

PNT was evaluated in in vivo models of mice with CML xenografted with Ba/F3 cells, which were transfected with both wild-type BCR-ABL1 and the BCR-ABL1^T315I^ mutation. The oral drug was administered to the mouse model expressing Ba/F3 BCR-ABL1 wild-type for 19 consecutive days. There was a significant increase in the mean survival rate of mice treated with 2.5 or 5 mg/kg PNT; these mice survived for 27.5 and 30 days, respectively (*p* < 0.01 for both doses), whereas vehicle-treated mice survived only 19 days after an intravenous injection of Ba/F3 cells. Furthermore, treatment with PNT increased the survival rates of mice injected with Ba/F3 cells expressing BCR-ABL1^T315I^. Treatment at different doses (5, 15, and 25 mg/kg) dose-dependently prolonged median survival (19.5, 26, and 30 days, respectively; *p* < 0.01 for all doses) compared with that of vehicle-treated mice, which survived for only 16 days [22].

In comparison, DST was administered at doses as high as 300 mg/kg and did not increase survival in mice that received injections of Ba/F3 cells expressing BCR-ABL1^T315I^, demonstrating its limited efficacy against the mutant enzyme. However, PNT has been shown to induce tumor stasis or regression in a subcutaneous BCR-ABL1^T315I^ mouse model at doses as low as 30 and 50 mg/kg. In summary, these initial results from preclinical studies confirmed its potential inhibitory effect against both native and mutant BCR-ABL1 cells [22,77].

PNT hydrochloride was approved by the FDA for oral use under the trade name Iclusig^®^ [78] as a treatment option for adult patients with the CML-positive T315I mutation, inaugurating the third generation of “tinibs” [78,79]. This medication is not limited to the chronic phase of the disease; it also demonstrates efficacy in the accelerated and blast phases. Moreover, it is indicated for patients with the T315I mutation and those with Philadelphia chromosome-positive (Ph+) acute lymphoblastic leukemia (ALL). These characteristics were observed in a phase II study known as PACE [80], which investigated the efficacy and safety of PNT in patients with CML and Ph+ ALL who demonstrated resistance or a lack of response to treatment with second-generation inhibitors (DST or NLT). This study also included patients with the T315I mutation [22]. The approval of PNT was given on 14 December 2012, with a focus on patients who demonstrated intolerance or resistance to two or more TKI inhibitors, such as DST, NLT, or IMT [22].

Additionally, the recent review by Kantarjian et al. contributes to the historical understanding of the medicinal chemistry and computational methods that founded the development of ponatinib. In this review, the authors thoroughly describe the preclinical and clinical trials that led to the regulatory approval of the drug, as well as discussing its current application in the treatment of CML and Ph+ ALL, comparing ponatinib to other third-generation BCR-ABL1 tyrosine kinase inhibitors [20]. For a comprehensive understanding of this inhibitor research and development process, it is recommended to read both reviews, as they cover the rationale behind each subunit of ponatinib’s chemical structure, as well as the progress of in vitro and in vivo trials. They also describe the serious adverse effects associated with the use of this potent inhibitor.

**Figure 19:**
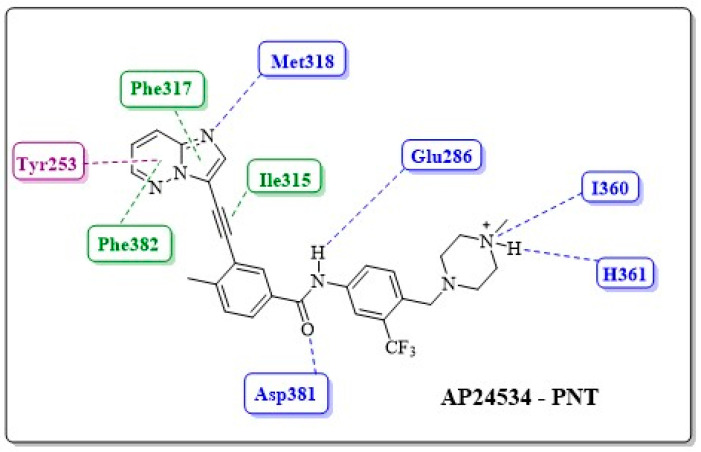
Two-dimensional interaction map between PNT (AP24534) and ABL1 residues (DFG-out conformation). The hydrogen bonds and van der Waals interactions are represented by blue and green dotted lines, respectively; putative pi-stacking interactions are represented by purple dotted lines [81].

Using the results of O’Hare et al., who cocrystallized PNT with the mutant enzyme ABL1^T315I^, Zhou et al. were able to evaluate the interactions of the inhibitor–enzyme complex with the aid of molecular modeling (Figure 19) [47,81].

From this work, it was possible to separate the moieties of PNT into six groups according to the regions in which they interact in the enzyme: the adeninomimetic hinge, gatekeeper linker 1, hydrophobic pocket ring A, DFG linker 2 interaction region, selective hydrophobic pocket ring B, and the region exposed to the residue–solvent terminal or tail.

✓ Adeninomimetic-hinge core (imidazolo[1,2-*b*]pyridazine): To mimic the adenine nucleus of ATP, fused heteroaromatic (nitrogenated) rings, culminating in imidazolo[1,2-*b*]pyridazine, were explored. The imidazole fits into the hinge region through a hydrogen bond-like interaction with Met318, establishing van der Waals interactions with residues Met318 and Phe317. The pyridazine ring establishes hydrophobic interactions with specific amino acids, such as Phe382 in the DFG triad and Tyr253 in the P-loop region (Figure 19) [81].
✓ Gatekeeper-linker 1 (ethynyl group): The SAR analysis of PNT revealed that the conformational restriction of the linker plays a crucial role in its ability to inhibit ABL1^T315I^. This may be due to steric relief to the gatekeeper residue (Ile315), allowing access to both the hinge region and the deeper region of the enzyme in the selective hydrophobic pocket provided by the inactive DFG-out conformation (as shown in Figure 17) [81].
✓ Hydrophobic pocket-ring A (arene moiety): This ring occupies the hydrophobic cavity of the BCR-ABL1 enzyme located behind the gatekeeper residue (Figure 19). The presence of the methyl group is essential because it allows access to the deeper region of the enzyme through a conformational restriction in the IMT, NLT, and PNT inhibitors. Changing positions or removing them can reduce their biological activity [81].
✓ DFG-linker 2 interaction region (amide group): This peptide bond between aromatic rings A and B results in crucial hydrogen bond-type interactions in the enzyme between the carbonyl oxygen of the amide and the Asp381 residue and the NH group with the Glu286 residue. These interactions anchor the inhibitor in the active site (Figure 19) [81].
✓ Selective hydrophobic pocket-ring B (arene moiety): This pocket-ring makes hydrophobic contact with the biomacromolecule. The presence of the trifluoromethyl group allows the exploration of new hydrophobic contacts in the deep region of the enzyme [81].
✓ Terminal residue or Tail (*N*-methylpiperazine moiety): Although the authors do not consider it one of the main fragments of PNT, crucial hydrogen bond-like interactions are established between the methylpiperazine group and His361 and Ile360 residues of the ABL1^T315I^ kinase structure (Figure 19) [81].

**Figure 20:**
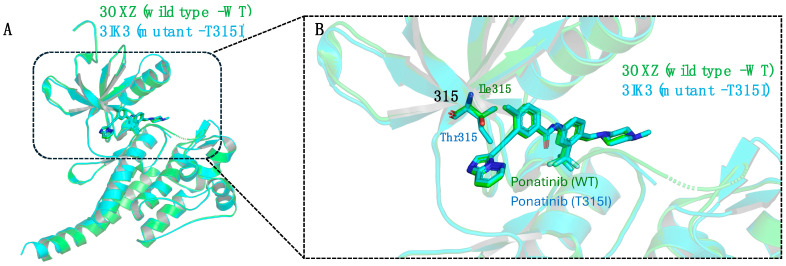
(**A**) Overlay between PNT complexes with native ABL1 (green) and ABL1^T315I^ (cyan), indicating the rotation of the N lobe about lobe C. (**B**) Overlay of PNT complexes with native ABL1 (green) and ABL1^T315I^ (cyan) [81].

Although there are no indications of steric conflicts in PNT-ABL1 complex interactions, the potency of the inhibitor decreases by approximately 5- to 7-fold in both enzymatic and cellular assays compared with its activity against wild-type ABL1, as shown in Figure 17 [81]. A reduction in the interaction of ring A in its hydrophobic site was observed compared with that of the native enzyme. The same phenomenon occurs with the B ring in the hydrophobic cavity in the C lobe if the ABL1 mutant protein maintains the same conformation as the native protein (Figure 20A) [81].

To clarify this observation, studies were conducted by Zhou et al. based on the native PNT-ABL1 complex and by O’Hare et al. on the cocrystallized PNT-ABL1^T315I^ complex (Figure 20A) [47,81]. In the case of the mutant enzyme, the isoleucine residue at position 315 would reduce the space of the catalytic orthosteric site, resulting in a potential steric clash with both the ethynyl spacer and the A ring of PNT (Figure 20B).

This hypothesis is based on the resistance observed in treatment with kinase inhibitors in cases of mutations at the native gatekeeper residue by an Ile or Met residue [82]. In the case of ABL1, this mutation becomes refractory to the main inhibitors, and there is an increase in kinase activity [83]. Xie et al., through NMR studies with the enzyme, have shown a shift in the conformational equilibrium toward the active form provided by the mutation [84]. Although the mechanism of action has not been elucidated for PNT, a molecular dynamics study performed by Ayaz et al. suggested a structural mechanism between IMT and wild-type BCR-ABL1 that could be related to PNT due to structural similarity and its role as an ATP-competitive inhibitor [85]. Through this study, they verified the selection of IMT by the autoinhibitory state (an inactive conformation) of the enzyme with subsequent induced fit. This understanding could inform a hypothesis about the differences observed in the case of the native and mutant BCR-ABL1.

Overlapping the C-terminal domains of the two kinases (mutant and native) reveals a subtle but visible twist in the N- and C-terminal domains of the ABL1^T315I^ kinase. Simultaneously, this twisting occurs with the displacement of the PNT out of the binding cavity (as illustrated in Figure 20B). As a result, the C domain is now deeply involved with the PNT B ring, establishing interactions similar to those observed in the native structure. This process involves crucial hydrophobic interactions between ABL1 and the CF_3_ group of ring B, which also form essential hydrogen bonds with the N-methyl piperazine group (Figure 20) [81].

The intrinsic flexibility of the kinase domain provides a crucial mechanism for maintaining the binding affinity of the DFG-out inhibitor to BCR-ABL1^T315I^. On the basis of this hypothesis, they associated the conformational adjustment of the protein, which generates a slight displacement of the A ring out of the hydrophobic site (as illustrated in Figure 20B), with a decrease in the potency of PNT against BCR-ABL1^T315I^, explaining the observed difference [81].

**Figure 21:**
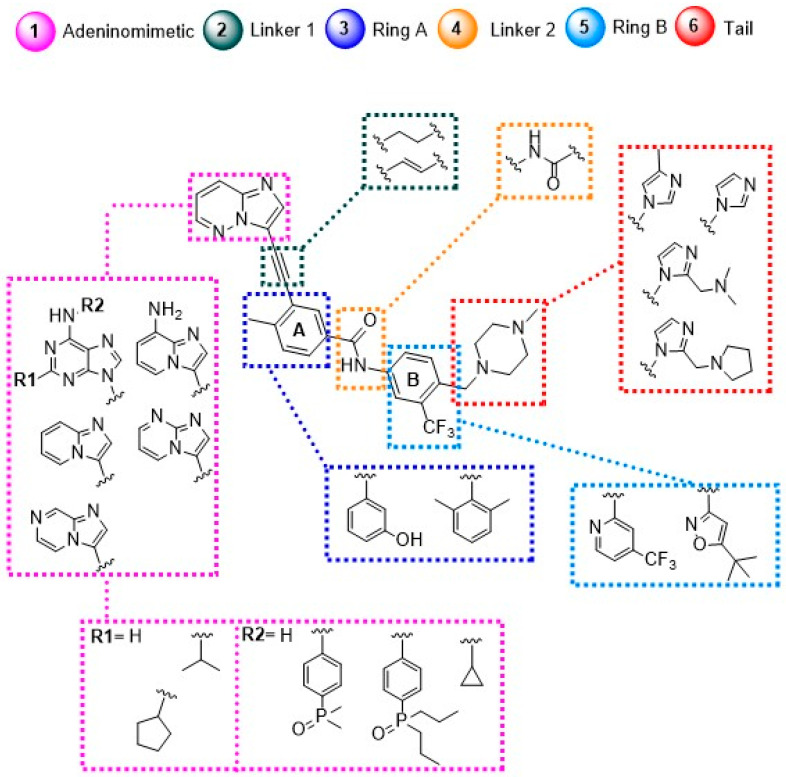
Schematic representation showing the main structural modifications involved in the development of the ponatinib structure [81].

A summary of the main modifications throughout the development process from prototypes to PNT is shown in Figure 21, as are the crucial regions of the enzyme’s orthosteric site.

## 2. Conclusions

Ponatinib (PNT) was developed as a third-generation tyrosine kinase inhibitor (TKI), effective in treating CML with the T315I mutation. This mutation confers resistance to first- and second-generation TKIs, such as IMT, DST, BST, and NLT, preventing effective binding to the BCR-ABL kinase domain. The development of PNT involved molecular modeling and X-ray crystallography to optimize interactions with the mutated domain, highlighting the importance of integrating drug design, clinical development, and regulatory review to deliver safe and effective therapies.

Approved by the FDA in 2012 for patients with CML resistance or intolerance to other TKIs, PNT demonstrated efficacy but faced challenges due to severe side effects, such as thrombosis and cardiotoxicity. These adverse events led to its temporary suspension and subsequent reintroduction with stricter guidelines.

The literature has yet to clarify which subunits of the PNT structure are responsible for its severe adverse effects. Nevertheless, PNT’s toxicity is preliminarily associated with its pan-inhibitory activity, meaning that it acts on several kinases, including those essential for cardiomyocyte survival, such as AKT and ERK. This broad inhibition suggests its high toxicity, which can result in severe and sometimes fatal adverse effects [37].

Despite the challenges, PNT has proven to be a crucial solution for treating resistant CML, representing a significant advance in oncological drug design. Its development exemplifies the importance of rational drug design and interdisciplinary collaboration in overcoming therapeutic resistance, pointing the way toward the creation of toward creating safer and more effective inhibitors.

## Figures and Tables

**Table 1:** IC_50_ (ABL1^T315I^) and cLogP values of AP23464 and its derivatives AP23848, AP23846, and AP23980 [42,47].

Compound	IC_50_ (ABL1^T315I^) (nM)	cLogP ^a^
**AP23464**	>5000	3.99
**AP23848**	5.1	5.36
**AP23846**	6.4	4.20
**AP23980**	297	4.62

^a^ Calculated with SwissADME [55].

**Table 2:** IC_50_ (ABL1^WT^) and cLogP values of AP23464, and its derivatives **1** and **2** [49].

Compound	IC_50_ (ABL1^WT^) ^a^ (nM)	cLogP ^b^
**AP23464**	61	3.99
**1**	15.8	4.67
**2**	3.58	3.68

^a^ GST-ABL1^WT^, peptide substrate [47]. ^b^ Calculated with SwissADME [55].

**Table 3:** IC_50_ (ABL1^WT^), IC_50_ (SRC), and cLogP values of AP23464, and its derivatives **2**, **3**, and **4** [23,49].

Compound	IC_50_ (ABL1^WT^) ^a^ (nM)	IC_50_ (SRC) (nM)	cLogP ^b^
**AP23464**	61	≤1	3.99
**2**	3.58	-	4.67
**3**	96	300	4.10
**4**	25	52	4.13

^a^ GST-ABL1^WT^, peptide substrate [47]. ^b^ Calculated with SwissADME [55].

**Table 4:** IC_50_ (K562, Ba/F3, ABL1^WT^, and SRC) and cLogP values of derivatives **4** and **5** [23].

Compound	IC_50_ (K562) ^a^	IC_50_ (Ba/F3) (BCR-ABL1^WT^) ^a^	IC_50_ (Ba/F3) (Parenteral) ^a^	IC_50_ (ABL1^WT^) ^a^	IC_50_ (SRC) ^a^	cLogP ^b^
**4**	67	47	>1000	25	52	4.13
**5**	95	57	7857	74	91	4.57

^a^ Concentration (nM). ^b^ Calculated with SwissADME [55].

**Table 5:** IC_50_ (ABL1^WT^) and IC_50_ (SRC) of compound **5** and its derivative **6** [23].

Compound	IC_50_ (ABL1^WT^) (nM)	IC_50_ (SRC) (nM)
**5**	74	91
**6**	23	74

**Table 6:** IC_50_ (Ba/F3, ABL1^WT^, ABL^T315I^, and SRC) and cLogP values of derivatives **6**, **7**, **8**, and **9** [23].

Compound	IC_50_ (Ba/F3) (BCR-ABL1^T315I^) ^a^	IC_50_ (Ba/F3) (Parenteral) ^a^	IC_50_ (ABL1^WT^) ^a^	IC_50_ (ABL1^T315I^) ^a^	IC_50_ (SRC) ^a^	cLogP ^b^
**6**	-	4353	23	-	11	3.92
**7**	-	4894	20	-	7	3.12
**8**	298	7120	13	542	8.0	4.36
**9**	422	6455	25	478	7.6	4.71

^a^ Concentration (nM). ^b^ Calculated with SwissADME [55].

**Table 7:** IC_50_ (ABL1^T315I^, Ba/F3, and ABL1^WT^) and cLogP values of AP24163 and its derivative **10** [23].

Compound	IC_50_ (ABL1^T315I^) ^a^	IC_50_ T315I (Ba/F3) ^a^	IC_50_ (ABL1^WT^) ^a^	cLogP ^b^
**AP24163**	478	422	25	3.99
**10**	386	295	1.6	3.41

^a^ Concentration (nM). ^b^ Calculated with SwissADME [55].

**Table 8:** IC_50_ (ABL1^WT^ and ABL1^T315I^) and cLogP values of derivatives **7**, **11**, **12**, and **13** [17,55].

Compound	IC_50_ (ABL1^WT^) ^a^	IC_50_ (ABL1^T315I^) ^a^	cLogP ^b^
**7**	20	14142	3.12
**11**	23	15513	3.74
**12**	30	524	3.32
**13**	28	359	3.78

^a^ Concentration (nM). ^b^ Calculated with SwissADME [55].

**Table 9:** IC_50_ (ABL1^WT^ and ABL1^T315I^) and oral bioavailability (F%) values of derivatives **15**, **16**, **17**, **18**, **19**, **20**, **21**, and **22** [17].

Compound	IC_50_ (ABL1^WT^) ^a^	IC_50_ (ABL1^T315I^) ^a^	F%
**15**	2.3	1216	5.5
**16**	26	102	42.4
**17**	45	168	ND
**18**	26	185	ND
**19**	9	56	29.0
**20**	69	639	ND
**21**	2.3	9	16.7
**22**	8.6	40	18.2

^a^ Concentration (nM). ND = not determined [17].
